# Editorial: Grappling with the Multifaceted World of the DNA Damage Response

**DOI:** 10.3389/fgene.2016.00091

**Published:** 2016-05-18

**Authors:** Antonio Porro

**Affiliations:** Institute of Molecular Cancer Research, University of ZurichZurich, Switzerland

**Keywords:** DNA damage, DNA damage response (DDR), DNA repair pathways

DNA is the repository of the genetic information in each living organism and its integrity and faithful transmission has to be ensured across generations for our own survival. Despite DNA has evolved as a more stable molecule than its ancient predecessor RNA, it is not able to guarantee life-long stability. Random changes occurring at the level of DNA represent the main source of genetic variability and the raw material on which Darwinian evolution acts.

In all organisms, cells experience massive amounts of damaging events each day. For instance, DNA injuries occur with a frequency of 10^4^–10^6^ in a single human cell per day. DNA lesions can have deleterious effects, as they interfere with basic cellular transactions, such as genome replication and transcription. If DNA injuries are mis-repaired or unrepaired, they may ultimately result in mutations or wider-scale genome aberrations that threaten cell homeostasis. As a proof of the fact, genome instability is a hallmark of tumorigenesis and tumor progression (Hanahan and Weinberg, 2011). On the other hand, DNA injuries increase during time as result of the imbalance between generation and scavenging of by-products deriving from cellular metabolism. Therefore, DNA damage promotes cellular senescence or cell death (Malaquin et al.), thus contributing to aging or to onset of aging-related disorders.

DNA damage can attack most parts of the DNA structure, ranging from minor and major chemical modifications, to single-strand breaks (SSBs) and gaps, to full double-strand breaks (DSBs; Brown and Baltimore, 2000). DNA lesions can arise as consequence of physiological processes like DNA replication (Jossen and Bermejo; Ouyang et al.) or can be caused by the exposure to environmental toxins. For example, the mis-incorporation of nucleotides during DNA replication contributes to the spontaneous mutation rate in an organism. While, canonical DNA polymerases are proofreading enzymes able to recognize and correct many of these errors, some mutations can escape this process. Other endogenously-arising DNA alterations lead to loss or modification of DNA bases (Lindahl, 1993). By-products of physiological cellular metabolism, such as reactive oxygen species (ROS) derived from oxidative respiration (Markkanen et al.), side-products of lipid peroxidation, or aldehyde metabolism (Finkel and Holbrook, 2000), constitute a permanent enemy to DNA integrity as they ultimately lead to DNA oxidation and breaks.

DNA damage is otherwise produced environmentally by chemical and physical sources. The most pervasive DNA-damaging agents are ultraviolet (UV) light derived from sunlight and ionizing radiation (IR). Despite the ozone layer absorbing the most dangerous part of the ultraviolet spectrum (UV-C), the other two types of UV radiation, UV-A and UV-B, are able to penetrate Earth's atmosphere and reach the planet's surface, thus being of greatest concern to humans. Exposure to UV radiation induces formation of cyclobutane pyrimidine dimers (CPDs) and 6–4 photoproducts. Such lesions distort DNA's backbone, introducing bends or kinks that can represent a serious impediment for transcription and replication processes. In addition, IR originating from the decay of naturally occurring radioactive compounds or from medical treatment employing X-rays and/or radiotherapy, also generates various forms of DNA damage. Finally, certain types of chemicals can also cause a variety of DNA lesions. DNA-damaging chemicals are mainly used in chemotherapy, but can be present in contaminated foods, such as heterocyclic amines produced in over-cooked meat or aflatoxins detected in contaminated peanuts. Remarkably, tobacco products derived by cigarette smoking are the most prevalent environmental DNA-damaging chemical agents as they cause a wide variety of DNA adducts and oxidation, which can ultimately trigger cancer of the lung and adjacent tissues.

In order to preserve the integrity of the genome, cells have evolved an integrated signaling network of damage detection and repair: the DNA damage response (DDR; Lindahl and Barnes, 2000). The DDR senses different types of genotoxic lesions and mounts coordinate and multi-faceted responses, that ultimately fix DNA lesions in a timely manner and prevent their conversion into permanent genome mutations (Hoeijmakers, 2001; Harrison and Haber, 2006; Harper and Elledge 2007; Jackson and Bartek, 2009). Moreover, the DDR also activate checkpoints to arrest or delay cell cycle progression or, if repair fails, trigger apoptosis. Cell cycle checkpoints are a genome surveillance mechanisms monitoring and controlling the timing and order of cell cycle events (Ferretti et al.; Jossen and Bermejo). Indeed, the DDR signaling pathways modulate the activity of cell cycle regulators and DNA repair enzymes, thus ensuring tight coordination of DNA repair with cell cycle progression. At molecular level, DDR is organized into an elaborate network of interacting pathways, the constituents of which can be grouped into three major classes of proteins that act in concert to translate signals of damaged DNA into appropriate downstream responses. These comprise (1) sensor proteins that recognize abnormally structured DNA and initiate the signaling response, (2) transducers factors that relay and amplify the damage signal on the surrounding chromatin structure, and to (3) effector proteins that ultimately lead to DNA damage repair (Bartek and Lukas, 2007; Harper and Elledge, 2007). Thus, the DDR necessitates to be spatiotemporally regulated (Ferrando-May et al.) because, if misused, it can wreak havoc on DNA integrity.

The wide diversity of DNA injuries requires the activation and cooperation of multiple and largely distinct DNA repair mechanisms (Stracker et al.). Different DNA damages are repaired by a sequence of catalytic events mediated by a plethora of enzymes. Currently DNA repair pathways can be grouped in different categories.

Nucleotide Excision Repair (NER), involves global genome repair (GGR) and transcription-coupled repair (TCR), recognizes and repair helix-distorting base lesions, such as pyrimidine dimers, induced by UV light. A key aspect of NER is the excision of the damaged DNA by specific endonuclease as a short oligonucleotide, thus leading to the formation of single-strand DNA, which is then acted by DNA polymerases before ligation occurs. In Base Excision Repair (BER), a non-helix distorting base modification, such as oxidation or alkylation, is recognized by a DNA glycosylase that initiates the excision of the modified base, thus leaving an apurinic or apirimidinic site, from which nuclease, polymerase and ligase enzymes can complete the repair. This pathway can operate via two sub-pathways, short-path (SP-BER) or long-path (LP-BER), based on the length of DNA re-synthesis. However, these two pathways often converge and cause the formation of a SSB, which is in turn sealed by a rapid process dependent on PARP [Poly (ADP-ribose) polymerase]-mediated signaling. In mismatch repair (MMR; Bak et al.; House et al.) incorrect polymerase proofreading or ribonucleotide mis-incorporation in the DNA chains occurring during DNA replication triggers the activation of post-replicative DNA repair machinery, which degrades mis-paired nucleotide of the newly synthesized strand, thus assisting DNA polymerases with another chance to generate an error-free copy of the template sequence (Jiricny, 2013). Notably, lesions that block replication forks progression are often by-passed by DNA damage tolerance (DTT) pathways. Specialized translesion synthesis (TLS) DNA polymerases, harboring a less stringent base-pair requirements than replicative polymerases, restart stalled replication forks, thus preventing their collapse and the consequent DSB formation, but at the expense of a higher mutation rate; DTT pathway promotes the completion rather than the accuracy of DNA replication. Repair of DSBs relays on two major pathways: non-homologous end joining (NHEJ; Lieber, 2008) and homologous recombination (HR; San Filippo et al., 2008). Whereas, NHEJ can operate throughout the cell cycle and is mostly used by post-mitotic cells, HR requires the presence of an undamaged homologous template, usually a sister chromatid, to mediate faithful repair and is restricted to S- and G2-phase of actively replicating cells. NHEJ promotes direct ligation of the two ends flanking the DSB without the need of extensive DNA-end processing. However, small insertions, deletions, and substitutions occurring at the break site, make NHEJ an error-prone process. On the contrary, HR mainly ensures an accurate repair of DSBs (Ferretti et al.; Guirouilh-Barbat et al.) as it uses the homologous chromosome as a template and it is often dedicated to fix breaks arising from DNA replication stress. HR requires the generation of ssDNA by DNA-end resection, which in turn invades the undamaged template leading to the formation of branched DNA structures. Therefore, DNA synthesis and recombination intermediate dissolution complete the HR-mediated repair process. Furthermore, DSBs which harbor a complementary flanking sequence can also be repaired by alternative end-joining (alt-NHEJ) also called microhomology-mediated end-joining (MMEJ), when microhomologies are present, or via single-strand annealing (SSA) when longer repeats are present (Decottignies). Although, MMEJ and SSA also rely on DNA-end resection reminiscent of HR, they can lead to loss of intervening sequence and thus are highly mutagenic (Kalan et al.; Guirouilh-Barbat et al.; Blanco and Matos). Lastly, DNA interstrand crosslinks (ICLs) represent the most serious kind of DNA lesions, as they must be repaired through a complex mechanism involving NER, TLS and HR, which are coordinated by the Fanconi Anemia (FA) pathway.

Spatiotemporal recruitment of DDR factors to sites of DNA damage is promoted by sensor proteins, which activate specific signaling cascades. It is becoming increasingly clear the biological relevance of chromatin structure and epigenetic marks in the DDR orchestration (Ferrando-May et al.; House et al.; Savic). Efficient repair of DNA damage is complicated by the fact that DNA is packaged into a condensed structure. Then, to facilitate access of the DNA repair machinery to the lesion, transient rearrangement of the chromatin has to occur. The nucleosome is the fundamental unit of the chromatin and consists of core particle, in which DNA is wrapped around a histone octamer. Various histone posttranslational modifications (PTMs) such as methylation, phosphorylation, acetylation, sumoylation, and ubiquitination have been reported at different amino acid residues of histones (Bartocci and Denchi; Bologna and Ferrari; Ouyang et al.; Pinder et al.; Vaz et al.). Thus, the large number of histone PTMs and the existence of diverse histone variants can define specific chromatin configurations, which characterize distinct stages in the DDR. Emerging evidences suggest that non-coding RNAs (ncRNAs), as master regulators of chromatin, can control the activation of DNA repair machinery by promoting chromatin organization in different epigenetic states. On the other side, ncRNAs, like microRNAs, may also act in the biogenesis of core protein-coding components of DDR pathways (Boucas et al.; Montecucco and Biamonti).

DDR regulates several physiological processes. DNA-repair enzymes can introduce physiological DSBs to promote genetic variability during meiosis in germ cells. DDR is indeed required to promote genetic diversity via sexual reproduction by ensuring the exchange of genetic information between homologous chromosomes before meiosis (Carroll and Marangos). Moreover, recombination processes are involved in the maturation of the immune system, such as class-switch and V(D)J recombination in B- and T-lymphocytes and play a critical role in the activation of immune surveillance and in generating immune-receptor diversity. Finally, DDR can determine whether a cell undergoes apoptosis or terminal differentiation through senescence. In this regard, markers of unrepaired DSBs accumulate with age at telomeres (Arnoult and Karlseder, 2015; Feuerhahn et al., 2015), which are nucleoprotein structures located at the end of our chromosomes. Due to the inability of the replication machinery to fully replicate chromosomal ends, telomeres shorten with each cell division until they hardly retain telomeric DNA repeats that are instead recognized as DSBs (Rosen; Henriksson and Farnebo). Thus, under such context of chronic DDR activation at telomeres, cells can enter into apoptosis or senescence (Fumagalli and d'Adda di Fagagna, 2009).

Considering the biological relevance of DDR in diverse physiological settings, “inherited” DDR defects predispose cells to genome instability and consequent diseases, like neurodegenerative disorders, immune deficiencies, infertility, age-related pathologies, cardiovascular diseases, metabolic syndromes, and cancer. The DDR is usually activated in precancerous cells experiencing oncogene-induced replicative stress and can be considered as an anticancer barrier that protects against full cellular transformation. Otherwise, targeting the replicative surge of cancer cells and their DDR/checkpoints unbalance are the basis for classical radio- and chemo-therapy and impairment of DNA repair pathways may represent a window for therapeutic opportunity (Shahbazi et al.; Kotsinas et al.). Cancer cells displaying specific DNA repair defects become “addicted” to complementary, but often inaccurate repair pathways in order to fuel their unscheduled expansion. Recently, this effect has been successfully exploited for synthetic lethality strategies, where small molecule inhibitors of these alternative pathways lead to selective killing of cancer cells harboring a specific genetic background, as in the case of PARP inhibitor treatment of HR-deficient tumors. Although, DDR components represent attractive targets for the development of novel cancer-therapies, they can also provide a common mechanism for cancer-therapy resistance. The development of diagnostic procedures to identify DDR components altered during oncogenesis might allow effective detection of pre-malignant diseases and tailor DNA-damaging or DDR inhibitor therapies for individual patients.

The relevance of the chemistry and biology of the DDR was underscored by the Royal Swedish Academy of Sciences when it awarded the Nobel Prize in Chemistry 2015 to three pioneering scientists-Thomas Lindahl, Paul Modrich, and Aziz Sancar-for having, independently of each-others, mapped and elucidated the molecular basis of how cells repair their DNA. In the early 1970s, Thomas Lindhal was the first scientist to demonstrate that the DNA decays at a slow but noticeable rate. This insight led him to quest for repair enzymes discovering in this way the BER pathway (Lindahl, 1974). At the same time, Aziz Sancar investigated the effects of UV radiation on bacteria leading him to uncover the NER pathway (Sancar and Rupp, 1983). Paul Modrich instead focused his research on DNA replication finding out how cell corrects errors during cell divisions: the mismatch repair mechanism (Lahue et al., 1989). In the last two decades, oncology research has been building on those findings to develop the aforementioned conventional DNA-damaging cancer treatment as well as newer targeted therapies by inhibiting repair pathways.

This Research Topic is aimed at comprehensive investigations of basic and novel mechanisms that underlie DNA damage response in eukaryotes. All authors in this Research Topic have provided their broad perspectives on distinct aspects of DDR and their insightful thoughts will benefit the field and will provide fertile ground for future investigations that we look forward to seeing develop.

## Author contributions

The author confirms being the sole contributor of this work and approved it for publication.

### Conflict of interest statement

The author declares that the research was conducted in the absence of any commercial or financial relationships that could be construed as a potential conflict of interest.
